# Visualized Emotion Ontology: a model for representing visual cues of emotions

**DOI:** 10.1186/s12911-018-0634-6

**Published:** 2018-07-23

**Authors:** Rebecca Lin, Muhammad “Tuan” Amith, Chen Liang, Rui Duan, Yong Chen, Cui Tao

**Affiliations:** 1 Krieger School of Arts & Sciences, Johns Hopkins University, Baltimore, MD USA; 2 School of Biomedical Informatics, The University of Texas Health Science Center, Houston, TX USA; 3 Health Informatics & Information Management, Louisiana Tech University, Ruston, LA USA; 4 Perelman School of Medicine, University of Pennsylvania, Philadelphia, PA USA

**Keywords:** Crowdsourcing, Emotion, Human computer interaction, Graphical user interfaces, Knowledge engineering, Knowledge representation, Public healthcare, Semantic web, Software agents

## Abstract

**Background:**

Healthcare services, particularly in patient-provider interaction, often involve highly emotional situations, and it is important for physicians to understand and respond to their patients’ emotions to best ensure their well-being.

**Methods:**

In order to model the emotion domain, we have created the Visualized Emotion Ontology (VEO) to provide a semantic definition of 25 emotions based on established models, as well as visual representations of emotions utilizing shapes, lines, and colors.

**Results:**

As determined by ontology evaluation metrics, VEO exhibited better machine-readability (*z*=1.12), linguistic quality (*z*=0.61), and domain coverage (*z*=0.39) compared to a sample of cognitive ontologies. Additionally, a survey of 1082 participants through Amazon Mechanical Turk revealed that a significantly higher proportion of people agree than disagree with 17 out of our 25 emotion images, validating the majority of our visualizations.

**Conclusion:**

From the development, evaluation, and serialization of the VEO, we have defined a set of 25 emotions using OWL that linked surveyed visualizations to each emotion. In the future, we plan to use the VEO in patient-facing software tools, such as embodied conversational agents, to enhance interactions between patients and providers in a clinical environment.

## Background

Emotions form the core of people’s thought processes, decisions, and actions, so it is crucial to investigate and understand them [1, 2]. In particular, some of the most highly emotional experiences for patients arise in healthcare scenarios regarding both acute and chronic illnesses. Clearly, the emotions felt in these situations are very complex, as evidenced by mixed emotions that may arise concerning a surgery—on one hand, hope that the surgery will successfully treat the patient’s disorder; on the other, fear that the surgery could fail and even jeopardize the patient’s life. A patient’s journey involves moving from sub-event to sub-event within one overarching emotion episode (for example, going from an emergency room visit to an inpatient hospital stay) in a state of continuous emotional engagement [3]. Unfortunately, these heightened emotions are likely to have a negative effect on patients, and influence the choices they make. For instance, patients experiencing high levels of anxiety tend to prefer safer (low-risk, low-reward) options, while patients experiencing high levels of sadness tend to prefer more comforting (high-risk, high-reward) options [4]. Moreover, patients often feel a sensation of powerlessness and lack of control over their bodies as well as their mental states, which may ultimately result in motivational, cognitive, and emotional deficits, and even depression [5]. These negative outcomes are reflected in the emotions of healthcare providers as well, who experience a significant amount of stress that may even increase their likelihood to commit malpractice [6].

On the other hand, fortunately, positive emotions initiate upward spirals toward enhanced emotional well-being [7]. Furthermore, patients who report higher levels of positivity tend to also participate more during health care service encounters [8], which is beneficial for all parties involved in a clinical experience, improving both perceived quality of service and customer satisfaction. Thus, this underscores the importance of promoting positive emotions in one’s patients. To do so, a phenomenon called emotional contagion, or “the tendency to automatically mimic and synchronize facial expressions, vocalizations, postures, and movements with those of another person and to converge emotionally” [9], can be utilized to invoke certain emotions—a healthcare provider could purposely express positive emotions so that the patient mirrors them. Additionally, emotional contagion has been confirmed by neural mechanisms, because an fMRI study has revealed that observing others’ happiness activates the left anterior cingulate gyrus, while observing others’ sadness activates the right inferior frontal gyrus [10]. Nonetheless, it does not make sense for physicians to remain positive all the time, as they often need to deliver upsetting diagnoses or prognoses, so communication skills training [11, 12] would be useful in teaching them how to give bad news while minimizing detrimental effects to a patient’s mental state. In any case, it is essential that healthcare providers can adequately understand and respond to their patients’ emotions to best ensure their well-being.

The first step in understanding emotions is to define what, exactly, an emotion is. According to Paul Ekman, emotions correspond to six universal facial expressions: joy, sadness, anger, disgust, fear, and surprise [13]. However, variations in response have undermined the reliability of using facial expressions to distinguish emotions, as well as using other characteristics such as skin conductance, heart rate variability, distinctive behaviors, patterns of feeling, and neuroimaging [14]. Rather, Ortony, Clore, & Collins’ models of emotions (“the OCC model”) differentiates 22 emotions depending on the psychological scenario that causes the emotion and the subsequent affective reactions that appear [15]. These affective reactions may include bodily, expressive, experiential, and behavioral responses—for example, the emotion “fear” is reflected by wide-eyed facial expressions and anxious thoughts that are caused by a threat.

The OCC model corresponds to a psychological constructivist approach for understanding emotion. According to this approach to the mind, discrete emotion categories are represented by general brain networks rather than localized ones corresponding to specific brain functions [16]. In fact, it is the interactions of domain-general networks like the salience network that cause different emotions to arise [17]. The OCC model is compatible with this constructivist approach because it proposes that emotions are comprised of a collection of behaviors rather than independent entities that then cause the behaviors. This model is especially helpful for our use, because describing emotions based on situations rather than on patterns of physiology, neurology, experience, expression, and motivation is more straightforward and reliable for computers to understand. Additionally, the OCC model organizes emotions into three categories: those concerning consequences of events, actions of agents, and aspects of objects. For instance, one can be happy or sad about a consequence (of an event), proud or ashamed about an action (of an agent), and love or hate an aspect (of an object). Additionally, in 2009, the emotions “interest” and “disgust” were added to the OCC model and its logical structure was changed so that it became inheritance-based [18]—this “revised OCC model” is what helped inform the structure for our own emotion model.

Even though the revised OCC model is inheritance-based and popular in the computer science realm [15], it has not yet been formally incorporated into a machine-readable artifact, so we decided to represent its information by constructing an ontological model. An ontology describes domain knowledge or domain space that represents and connects concepts of the domain. These concepts and relationships can be encoded to a machine language using semantic web coding languages (e.g., OWL and RDF), thereby allowing machines to process and understand the domain knowledge. The resulting software artifact can then be integrated with other software components to provide extended capabilities, perform tasks, and enable machine reasoning.

Thus, the first purpose of our Visualized Emotion Ontology (VEO) is to semantically define emotions based on the Ekman [13] and revised OCC [18] models in a machine-readable artifact; the second purpose of the VEO is to create visualizations for each of the 25 emotions 1 in our model by connecting them to shapes, lines, and colors.

To investigate the relationship between emotions and shapes, Bar and Neta [19] asked subjects to rate pictures of everyday objects (e.g., a watch or a sofa) with either curved or angular features, finding that participants liked curved objects significantly more than angular objects. Similarly, other studies have found that humans associate circles with positive emotions and triangles with negative emotions [20, 21]. In particular, humans find triangles with downward-pointing vertices to be the most unpleasant shape, because in comparison to triangles with upward-pointing vertices, viewing downward-pointing triangles resulted in significantly higher levels of activation in the threat detection areas of the brain such as the amygdala, subgenual anterior cingulate cortex (ACC), superior temporal gyrus (STG), and fusiform gyrus [22]. One plausible explanation is that these shapes mirror human facial features–when people are happy, their facial expressions naturally appear rounder, but when people are angry, their facial expressions appear more angular, much like a downward-pointing triangle [20]. Moreover, mirroring the findings about emotions and shapes, studies about emotions and lines have established that curved lines evoke a positive response while sharp lines evoke a negative response [19], and that a greater number of lines provokes a stronger response [20].

In terms of the relationship between emotions and colors, in one study, when participants were asked to categorize anger and sadness words presented in red or blue, they categorized anger words faster and more accurately when the font color was red rather than blue, and vice versa for sadness words [23]. Multiple other studies have confirmed that the color red is associated with anger [24–27] and danger [28], though it is also associated with romance [24, 29]. Additionally, people identify yellow with happiness [24, 25] and orange with cheerfulness [25], though they associate blue with sadness [23–25] as well as calmness [24]. Green is linked to success [30] and safety [28], but disgust as well [24, 25]. Brown is also associated with disgust [25] and white is connected to innocence and hope [24], while purple and black are both linked to power, contempt, sadness, and fear [24, 25].

Thus, the VEO serves as a machine-understandable artifact with human-friendly visualizations; as such, one of the future directions of this work is towards human-computer integration. The focus on using situations to define emotions in the revised OCC model could help computers understand how different emotions arise and provide some artificial emotional intelligence to machines. In the next section, we discuss some applications for emotion-related ontologies; however, the aim, aside from modeling the emotion domain and visualizations, is to incorporate our emotion images into embodied conversational agents as an alternative to more complex virtual facial features and to create an ontology-driven “face-plate”, specifically for use in healthcare applications.

Overall, we assert that 1) *we can faithfully represent a high quality ontological artifact of the OCC model of emotion using a semantic web language (OWL2) that links evidence-based visualized cues for each defined emotion*, and 2) *that the aforesaid visualizations can accurately symbolize each emotion defined in our ontology*. For the first assertion, we will evaluate the ontology using the Burton-Jones’ semiotic metric suite that measures quality based upon dimensions from semiotic theory. The ratings will be produced by Ontokeeper, and we will compare the results with other cognitive ontologies. For the second, we will use a survey submitted through a crowdsourcing platform to gauge the symbolic visualizations of emotions.

This paper extends on our previous work, introduced in [31] where we briefly discussed the design of the VEO. In this paper, we expound on the detailed design motivations behind the VEO and its linked visualizations, and in addition, we provided an evaluation of the ontology using Burton-Jones’ semiotic metric suite and validated the visualizations using a crowdsourcing platform.

### Related studies on emotion ontologies and visualization

The Human Emotion Ontology (HEO) by Grassi [32] was an ontology aimed at annotating emotions in multimedia content. Developed in OWL, the central concept of the ontology was *Emotion* which incorporated components of emotions described by the W3C Emotion Incubator Group [33]. Also, HEO models concepts and ideas from Ekman’s and Douglas-Cowie’s classification of emotions, the actions related to emotions by [3] and Scherer’s appraisal model [34]. It also represents the modality of the emotion, ranging from voice, text, gesture, and face. At the time of publication, HEO is not publicly available, and there is no evidence of further updates since the 2009 publication.

An ontology that converges on similar ideas as the VEO is the Smiley Ontology [35] for “representing the structure and semantics" of an emoticon. In their ontology model, each emoticon is associated with an emotion, and the emoticon is further defined by concepts concerning the verbal features of the emoticon, the textual context, analogous human facial expression, etc. Like HEO, the Smiley Ontology is no longer active. Another important work involves Garcia-Rojas and colleagues’ use of ontology to semantically annotate MPEG-based facial animation characteristics for virtual human characters [36]. While not an emotion ontology, WN-AFFECT [37] is an extension of the WordNet ontology with annotations that describe the emotional valence of words based on the W3C lists of emotions.

The Emotion Ontology (EMO) [38] is another formal representation for emotions that related affective phenomena and is aligned with the Basic Formal Ontology (BFO) [39, 40] and the Ontology of Mental Disease (OMD) [41], which allows it to express philosophical concepts. It distinguishes “emotions proper”, such as anger and fear, from appraisals (cognitive judgments, e.g., “appraisal of dangerousness”) and subjective feelings (inner awareness of affective feelings, e.g., “feeling restless”) [42]. We decided to align the VEO with the EMO, though we chose not to use all of the emotions in EMO because our model is more concise in regard to the number of emotions it includes, leaving out behavioral and cognitive responses that are not technically emotions, such as confusion, boredom, and guilt. Rather than being emotions themselves, they would appear in response to an emotion; for instance, “guilt” would stem from the emotion “shame”.

Additionally, one research group utilized visualizations to model emotions by developing a mobile messaging system called eMoto for users to send and receive affective messages [43]. Users navigated a circular background of colors, shapes, and animations where the vertical axis indicates arousal (moving upward corresponds to increasing arousal, from a few slow animations to many fast animations) and the horizontal axis indicates valence (moving right corresponds to increasing positive valence, from blue-purple-red to green-yellow-orange and from sharper shapes to rounder shapes). Compared to the VEO, eMoto was driven by the user’s interpretation of the emotions of their message, so it was much more fluid in both the types of shapes and the spectrum of hues that it uses, whereas the VEO provides fixed combinations of colors and shapes representing specific emotions.

## Methods

### Development of the Visualized Emotion Ontology

**Fig. 1:**
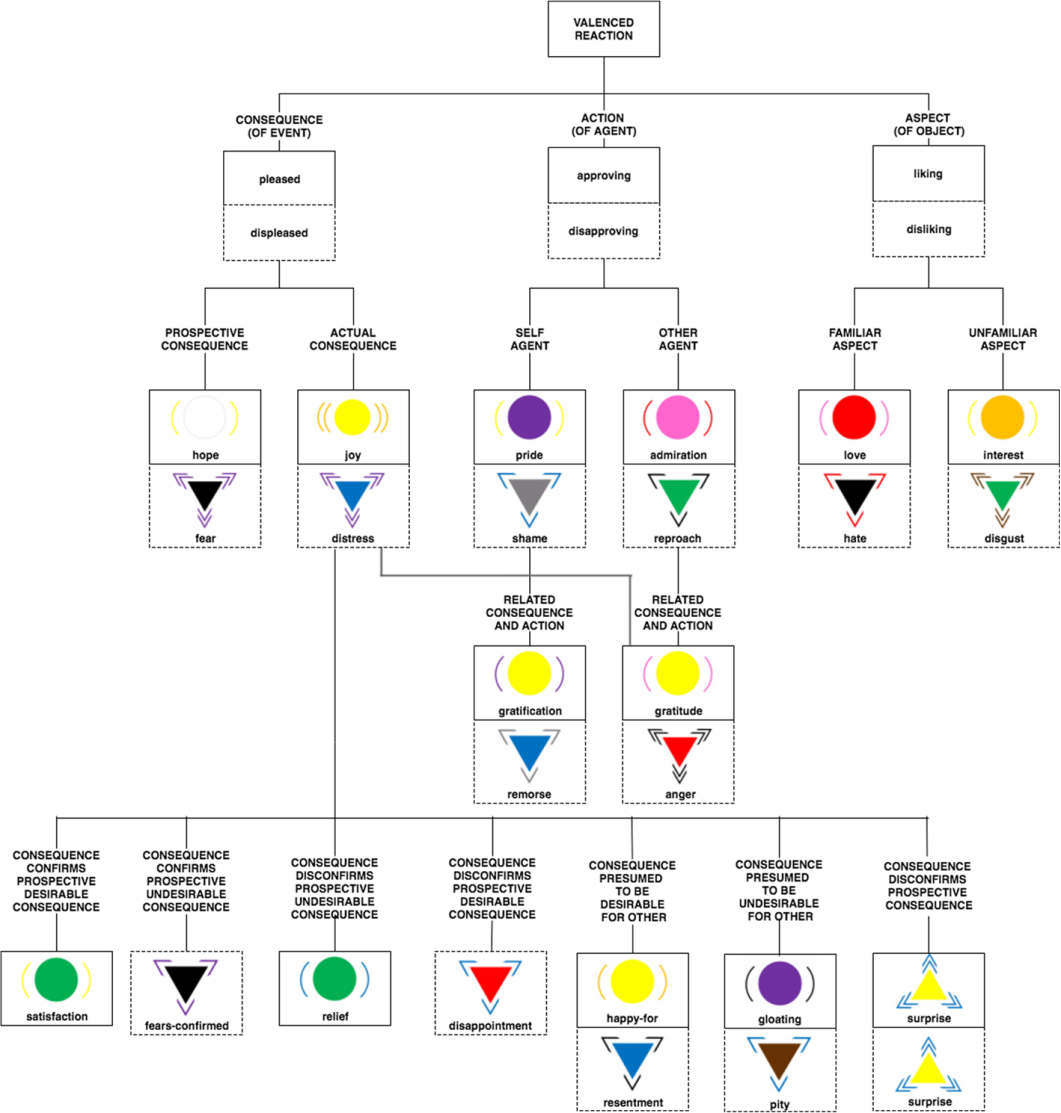
The VEO model of emotions framed from [15, 18]. The boxes with solid lines are of positive valence and the boxes with dotted lines are of negative valence

We designed the Visualized Emotion Ontology (VEO) that is organized on the revised OCC model, pairing the positive (solid-lined boxes) and negative emotions (dotted-lined boxes) (Fig. 1).

**Fig. 2:**
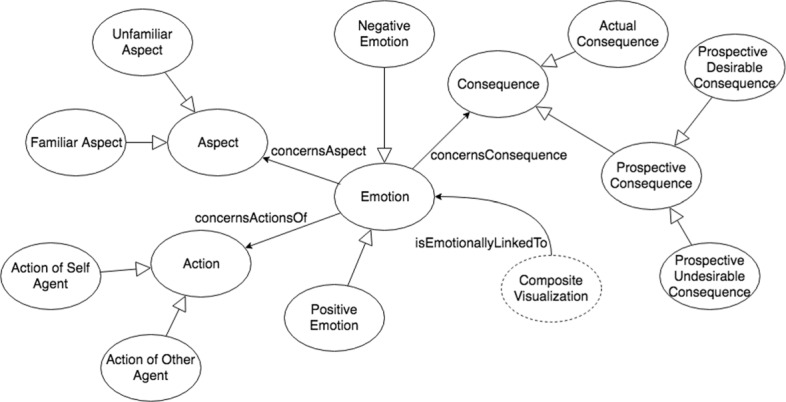
Brief class level conceptualization of the VEO

Our ontology is defined as a polyarchy with five branches, including *Action*, *Aspect*, *Consequence*, *Emotion*, and *Visualization* (Fig. 2). An *Action* is defined as either an *Action of Self Agent* or an *Action of Other Agent*, an *Aspect* is defined as either a *Familiar Aspect* or *Unfamiliar Aspect* of an object, and a *Consequence* is defined as either a *Prospective Consequence* or an *Actual Consequence* of an event. A *Prospective Consequence* can be further divided into *Prospective Desirable Consequence* or *Prospective Undesirable Consequence*, and an *Actual Consequence* can be further divided into a *Consequence Desirable for Other* or a *Consequence Undesirable for Other*, as well as a *Confirmed Consequence* or *Disconfirmed Consequence*. These terms are all in accordance with the revised OCC model [18]. As an example, a person would feel relief when a prospective undesirable consequence is disconfirmed, and in our model, that would be represented as a *Disconfirmed Undesirable Consequence*. Similarly, a person feels happy for another person when the other person experiences a desirable consequence, which we express as a *Consequence Desirable for Other*.

An *Emotion* is divided into either a *Positive Emotion* or a *Negative Emotion* subclass, which then can be further divided into *Approving*/*Disapproving*, *Liking*/*Disliking*, and *Pleased*/*Displeased* subclasses, respectively. Then, emotions are categorized into one or more of these subclasses in accordance with the revised OCC model. Beyond being defined hierarchically, they are defined further semantically. For instance, the emotion *Joy* is a subclass of *Pleased* and inherits the property *concernsConsequence*, but clarifies that the type of *Consequence* the property describes is an *Actual Consequence*; following this, the emotion *Satisfaction*, which is a subclass of *Joy*, further classifies the type of *Actual Consequence* as a *Confirmed Desirable Consequence*. Similarly, the emotion *Gloating* is also a subclass of *Joy*, but the type of *Actual Consequence* that it concerns is a *Consequence Undesirable for Other*. As another example, the emotion *Anger* is a subclass of both *Distress* and *Reproach*, which are subclasses of *Displeased* and *Disapproving*, respectively, so it inherits both properties of concerning an *Actual Consequence* and an *Action of Other Agent*. Finally, to show an example for the *Liking*/*Disliking* branch, the emotion *Love* inherits the property *concernsAspect* of a *Familiar Aspect*.

**Fig. 3:**
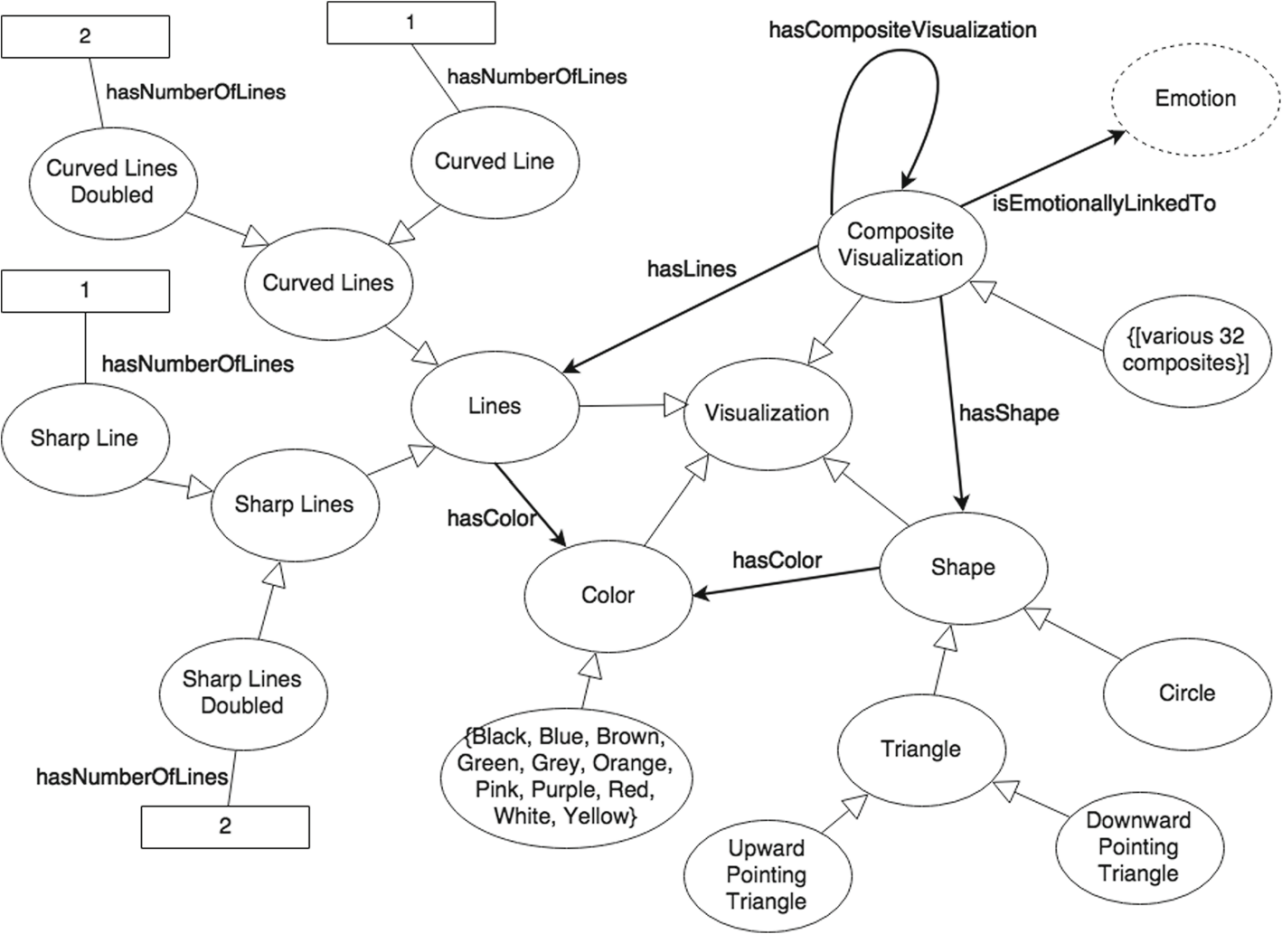
Concepts of *Visualization* classes from the VEO

Finally, *Visualization* (see Fig. 3) contains the subclasses *Color*, *Shape*, *Lines*, and *Composite Visualization*. The *Color* class involves *Black*, *Blue*, *Brown*, *Green*, *Grey*, *Orange*, *Pink*, *Purple*, *Red*, *White*, and *Yellow*; the *Shape* class includes *Circle* and *Triangle*, which can be either a *Downward Pointing Triangle* or an *Upward Pointing Triangle*; and the *Lines* class consists of *Curved Lines* and *Sharp Lines*. Within the *Curved*/*Sharp Lines* classes, we defined two subclasses *Curved*/*Sharp Line* and *Curved*/*Sharp Lines Doubled*, which have the data property *hasNumberOfLines* with value 1 and 2, respectively. Ultimately, these three subclasses of *Visualization* allowed us to create the *Composite Visualization* class, which combines a *Color* and a *Shape* or a *Color* and *Lines* to create such visualizations as *Yellow Circle* and *Black Sharp Line* by using the object properties *hasColor*, *hasShape*, and/or *hasLines*. Furthermore, *Composite Visualization* has an association with one *Emotion* with an object property called *isEmotionallyLinkedTo*. This allows us to define individual emotion visualizations, such as *Admiration Visualization*, which is a combination of a *Pink Circle* and a *Red Curved Line* that is linked to the emotion *Admiration*.

We defined the *Emotion* class in the VEO as equivalent to the *emotion process* class in EMO as well as any emotions that overlapped between the two ontologies, though we must recognize that there are emotions listed in our ontology that are not in EMO (e.g., “happy-for”) and vice versa (e.g., “boredom”). The *Emotion* classes equivalent between the VEO and the EMO were *Positive Emotion*, *Pride*, *Interest*, *Pleased* (*pleasure*), *Hope*, *Joy* (*happiness*), *Negative Emotion*, *Shame*, *Disgust*, *Hate*, *Distress* (*sadness*), *Anger*, *Disappointment*, *Fear*, and *Surprise*. Additionally, the *Action* class in the VEO was set as equivalent to the *behavior* and *behavior process* classes in EMO. For each emotion in the VEO, we included a definition, a description of the visualization, as well as a link to an actual image. Our initial version of the VEO is available here: https://bioportal.bioontology.org/ontologies/VEO.

### Development of visualizations for emotions

Next, in terms of visualizing the emotions, we combined results from current literature about emotions and their relationships with colors, shapes, and lines to create a unique visualization for each emotion (Table 1). The visualizations were created on Microsoft Word using standard colors (with the exception of pink, brown, and grey) and basic shapes. We decided to use two colors for each emotion–one color for the shape and one for the lines–because it is possible for a color to have either a positive or a negative connotation (e.g., red can represent anger or romance/love [24, 27–29]), so using more colors will help pinpoint the emotion that the visualization is supposed to represent. This would also ensure that no two emotions have the same visualization. However, it is important to recognize that not all of the emotions in the revised OCC model have yet been examined by other studies and linked to exact colors (e.g., pride), but in these instances, we made assumptions based on the connotations of the color and emotion. All of the positive emotions (e.g., joy) were portrayed as circles surrounded by curved lines and all of the negative emotions (e.g., distress) were portrayed as downward-pointing triangles surrounded by sharp lines. Also, after noticing that most of the emotions in the Ekman model overlapped with those in the OCC model (joy, distress, anger, disgust, fear), we indicated those emotions by doubling the lines surrounding the shape to increase their perceived significance.

**Table 1:** Visualization motifs for emotions

Emotion	Shape attribute	Line attribute
Admiration	Pink circle	Curved red lines, single
Anger	Red downward triangle	Curved black lines, double
Disappointment	Red downward triangle	Sharp blue lines, single
Disgust	Green downward triangle	Sharp brown lines, double
Distress	Blue downward triangle	Sharp purple lines, doubled
Fear	Black downward triangle	Sharp purple lines, doubled
Fears-confirmed	Black downward triangle	Sharp purple lines, single
Gloating	Purple circle	Curved black lines, single
Gratification	Yellow circle	Curved purple lines, single
Gratitude	Yellow circle	Curved pink lines, single
Happy-For	Yellow circle	Curved orange lines, single
Hate	Black downward triangle	Sharp red lines, single
Hope	White circle	Curved yellow lines, single
Interest	Orangle circle	Curved yellow lines, single
Joy	Yellow circle	Curved orange lines, doubled
Love	Red circle	Curved pink lines, single
Pity	Brown downward triangle	Sharp blue lines, single
Pride	Purple circle	Curved yellow lines, single
Relief	Green circle	Curved blue lines, single
Remorse	Blue downward triangle	Sharp gray lines, single
Reproach	Green downward triangle	Sharp black lines, single
Resentment	Blue downward triangle	Sharp black lines, single
Satisfaction	Green circle	Curved yellow lines, single
Shame	Gray downward triangle	Sharp blue lines, single
Surprise	Yellow upward triangle	Sharp blue lines, double

Thus, joy is visualized as a yellow circle surrounded by double curved orange lines due to the association of the color yellow with happiness and orange with cheerfulness [24, 25]. Distress, anger, disgust, and fear are all depicted as downward-pointing triangles surrounded by double sharp lines, with colors of the triangles and lines as blue and purple, red and black, green and brown, and black and purple, respectively. Both blue and purple are associated with sadness, red and black with anger, green and brown with disgust, and black and purple with fear [24, 25]. Hupka et al. [44] found that even across cultures (Germany, Mexico, Poland, Russia, and the United States), people associate anger with black and red, fear with black, and jealousy with red.

Though surprise is not in the revised OCC model, it is in the Ekman model, so we decided to add it as an emotion in our VEO model with the property that it arises when a consequence disconfirms a prospective consequence. However, an interesting issue arises because people can experience surprise in either a positive or negative context–for instance, in the workplace, receiving a raise would be a good surprise, while getting laid off would be a bad surprise. So in the VEO model, we decided to include surprise as both a positive or negative emotion: as a subclass of both joy and distress. Due to these two parent classes, we expressed its colors as yellow and blue [25], and its shape as an upward-pointing triangle (because its valence is between that of a circle and a downward-facing triangle) [22]. Thus, its complete visualization is a yellow upward-pointing triangle with double sharp blue lines.

Naturally, positive emotions are linked to joy, so the color yellow appears often in the visualizations for other positive emotions as well. For example, happy-for is visualized as a yellow circle surrounded by curved orange lines, which is the same color and shape combination as joy, except the lines are not doubled, indicating that joy is the “stronger” of the two emotions. Interest is depicted as an orange circle surrounded by curved yellow lines, which also indicates a sense of cheerfulness and joy, but the circle being orange rather than yellow lends a sense of unfamiliarity to the visualization (since interest is liking an unfamiliar aspect of an object) (See Table 2). Next, hope is portrayed as a white circle surrounded by curved yellow lines due to the association of white with hope and yellow with joy.

**Table 2:** Definition of positive emotions

Definition	OWL2 Axiom
Positive is a valenced reaction (to “something”)	Positive_Emotion $ \sqsubseteq $ Emotion
Pleased is being positive about a consequence (of an event)	Pleased $\sqsubseteq $ Positive_Emotion ⊓ (∃ concernsConsequence. Consequence)
Hope is being pleased about a prospective consequence (of an event)	Hope $\sqsubseteq $ Pleased ⊓(∃ concernsConsequence. Prospective_ Consequence)
Joy is being pleased about an actual consequence (of an event)	Joy $ \sqsubseteq $ Pleased ⊓ (∃ concernsConsequence.Actual_ Consequence)
Satisfaction is joy about the confirmation of a prospective desirable consequence	Satisfaction $\sqsubseteq $ Joy ⊓ (∃ concernsConsequence. Confirmed_Desirable_ Consequence)
Relief is joy about the disconfirmation of a prospective undesirable consequence	Relief $ \sqsubseteq $ Joy ⊓ (∃ concernsConsequence.Disconfirmed_ Undesirable_Consequence)
Happy-for is joy about a consequence (of an event) presumed to be desirable for someone else	Happy_For $ \sqsubseteq $ Joy ⊓ (∃ concernsConsequence. Consequence_Desirable_For_Other)
Gloating is joy about a consequence (of an event) presumed to be undesirable for someone else	Gloating $ \sqsubseteq $ Joy ⊓ (∃ concernsConsequence. Consequence_Undesirable_For_Other)
Surprise is joy or distress about the disconfirmation of a prospective undesirable consequence	Surprise $ \sqsubseteq $ Joy ⊓ (∃ concernsConsequence. Disconfirmed_Consequence) Surprise $ \sqsubseteq $ Distress ⊓ (∃ concernsConsequence. Disconfirmed_Consequence)
Approving is being positive about an action (of an agent)	Approving $ \sqsubseteq $ Positive_Emotion ⊓ (∃concernsActionsOf.Action)
Pride is approving of one’s own action	Pride $ \sqsubseteq $ Approving ⊓ (∃ concernsActionsOf. Action_Of_Self_Agent)
Admiration is approving of someone else’s action	Admiration $ \sqsubseteq $ Approving ⊓ (∃ concernsActionsOf.Action_Of_ Other_Agent)
Gratification is pride about an action and joy about a related consequence	Gratification $ \sqsubseteq $ Pride Gratification $ \sqsubseteq $ Joy (n.b.)
Gratitude is admiration about an action and joy about a related consequence	Gratitude $ \sqsubseteq $ Admiration Gratitude $ \sqsubseteq $ Joy
Liking is being positive about an aspect (of an object)	Liking $ \sqsubseteq $ Positive_Emotion ⊓ (∃ concernsAspect.Aspect)
Love is liking a familiar aspect (of an object)	Love $ \sqsubseteq $ Liking ⊓ (∃ concernsAspect.Familiar_Aspect)
Interest is liking an unfamiliar aspect (of an object)	Interest $ \sqsubseteq $ Liking ⊓ (∃ concernsAspect.Unfamiliar_Aspect)

Furthermore, we illustrated pride as a purple circle surrounded by curved yellow lines and gloating as a purple circle surrounded by black lines because purple has the connotations of arrogance and power, corresponding to both pride and gloating. However, pride is taking joy in one’s own accomplishments, so we used yellow as a complementary color to purple to express the relative positivity of this emotion, whereas gloating is taking joy in another’s misfortunes, so we used black as a complementary color to the purple to express the relative negativity of that emotion. Similarly, gratification is visualized as a yellow circle surrounded by curved purple lines, combining the colors of the visualizations of joy and pride in accordance to its definition (Table 2).

Next, love is presented as a red circle surrounded by curved pink lines due to the color red’s connection with romance [24, 29]. The color pink is technically a lighter shade of red created from mixing red and white, so by extension, it is also connected to romance. Due to this, we depicted admiration as a pink circle surrounded by curved red lines because it is very similar to love, while at the same time, possessing more emotional distance and less romantic feelings than love. Likewise, gratitude is portrayed as a yellow circle surrounded by curved pink lines, combining the colors of the visualizations of joy and admiration in accordance to its definition (Table 2). Additionally, satisfaction is illustrated as a green circle surrounded by curved yellow lines due to the association between green and success [30] as well as yellow and joy. Meanwhile, relief is illustrated as a green circle surrounded by curved blue lines due to the association between green and safety [28] as well as blue and calmness [24].

As for negative emotions, we depicted fears-confirmed as a black downward-pointing triangle surrounded by sharp purple lines, which is the same color and shape combination as fear, except the lines are not doubled, indicating that fear is the “stronger” of the two emotions. Next, hate is portrayed as a black downward-pointing triangle surrounded by sharp red lines due to the association of black and red with fear, anger, and a sense of evil. Consequently, reproach is presented as a green downward-pointing triangle surrounded by sharp black lines due to the connections with green and disgust and black and hate. Then, we characterized pity as a brown downward-pointing triangle surrounded by sharp blue lines due to the feelings it evokes of disgust and distress. Additionally, we characterized disappointment as a red downward-pointing triangle surrounded by sharp blue lines due to the association with red and failure [28, 30] as well as blue and sadness.

In addition, many negative emotions are related to distress (See Table 3 for negative emotion definitions), so blue is a prominent color among these visualizations. For instance, resentment is depicted as a blue downward-pointing triangle surrounded by sharp black lines due to its connotations of distress and contempt toward another person. Shame is presented as a grey downward-pointing triangle surrounded by sharp blue lines because both grey and blue have associations with sadness and depression [24, 25], but using grey also represents the contempt toward oneself that shame evokes. Similarly, remorse is presented as a blue downward-pointing triangle surrounded by sharp grey lines, with a reversed color and shape combination because it is derived from shame but places more emphasis on sorrow than on self-hatred.

**Table 3:** Definition of negative emotions

Definition	OWL2 Axiom
Negative is a valenced reaction (to “something”)	Negative_Emotion $ \sqsubseteq $ Emotion
Displeased is being negative about a consequence (of an event)	Displeased $ \sqsubseteq $ Negative_Emotion ⊓ (∃ concernsConsequence.Consequence)
Fear is being displeased about a prospective consequence (of an event)	Fear $ \sqsubseteq $ Displeased ⊓ (∃ concernsConsequence.Prospective_Consequence)
Distress is being displeased about an actual consequence (of an event)	Distress $ \sqsubseteq $ Displeased ⊓ (∃ concernsConsequence.Actual_Consequence)
Fears-confirmed is distress about the confirmation of a prospective undesirable consequence	Fears_Confirmed $ \sqsubseteq $ Distress ⊓ (∃ concernsConsequence.Confirmed_Undesirable_ Consequence)
Disappointment is distress about the disconfirmation of a prospective desirable consequence	Disappointment $ \sqsubseteq $ Distress ⊓ (∃ concernsConsequence.Disconfirmed_Desirable_ Consequence)
Resentment is distress about a consequence (of an event) presumed to be desirable for someone else	Resentment $ \sqsubseteq $ Distress ⊓ (∃ concernsConsequence.Consequence_Desirable_ For_Other)
Pity is distress about a consequence (of an event) presumed to be undesirable for someone else	Pity $ \sqsubseteq $ Distress ⊓ (∃ concernsConsequence.Consequence_Undesirable_For_Other)
Surprise is joy or distress about the disconfirmation of a prospective undesirable consequence	Surprise $ \sqsubseteq $ Joy ⊓ (∃ concernsConsequence.Disconfirmed_Cosnsequence) Surprise $ \sqsubseteq $ Distress ⊓ (∃ concernsConsequence.Disconfirmed_Consequence)
Disapproving is being negative about an action (of an agent)	Disapproving $ \sqsubseteq $ Negative_Emotion ⊓ (∃ concernsActionsOf.Action)
Shame is disapproving of one’s own action	Shame $ \sqsubseteq $ Disapproving ⊓ (∃ concernsActionsOf.Action_Of_Self_Agent)
Reproach is disapproving of someone else’s action	Reproach $ \sqsubseteq $ Disapproving ⊓ (∃ concernsActionsOf.Action_Of_Other_Agent)
Remorse is shame about an action and distress about a related consequence	Remorse $ \sqsubseteq $ Shame Remorse $ \sqsubseteq $ Distress
Anger is reproach about an action and distress about a related consequence	Anger $ \sqsubseteq $ Reproach Anger $ \sqsubseteq $ Distress
disliking is being negative about an aspect (of an object)	Disliking $ \sqsubseteq $ Negative_Emotion ⊓ (∃ concernsAspect.Aspect)
Hate is disliking a familiar aspect (of an object)	Hate $ \sqsubseteq $ Disliking ⊓ (∃ concernsAspect.Familiar_Aspect)
Disgust is disliking an unfamiliar aspect (of an object)	Disgust $ \sqsubseteq $ Disliking ⊓ (∃ concernsAspect.Unfamiliar_Aspect)

### Surveys

**Fig. 4:**
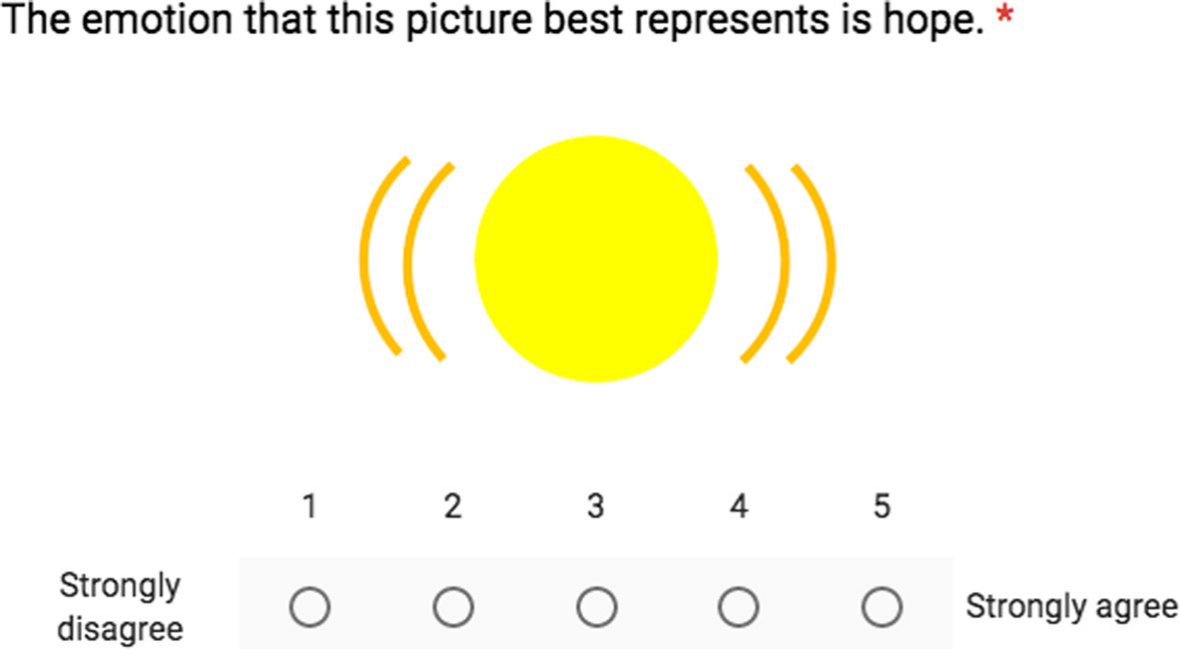
Example of a survey question for hope visualization

We conducted surveys^2^ to validate and assess our visualizations of emotions that we designed for adult participants (*n* = 1082) of any gender residing in the United States, recruited through Amazon Mechanical Turk (MTurk). Studies have shown that data obtained from MTurk are at least as reliable as those obtained via traditional methods [45]. Using Qualtrics, we created a 51-question survey involving our 25 distinct emotions, in which we asked MTurk participants to rate the validity of a statement matching an emotion to an image based on our model. The incorrect emotion-image pairs were selected randomly from the 24 other emotions in our model. For instance, the word “distress” displayed with our visualization for “distress” would be a correctly-matched emotion-image pair, but the word “distress” displayed with our visualization for “fear” would be an incorrectly-matched pair. Finally, we included one randomly placed control question in each survey (e.g., “So we can be sure that you are reading the questions carefully, please answer ’Strongly agree’ to this question.”) to identify and remove participants who rushed through the survey. Each MTurk Human Intelligence Task (HIT) included one assignment with a link to this Qualtrics survey; the HIT was launched from August 5-14, 2017, and the reward was $0.20 per assignment. In total, 1189 people completed the HIT, but 107 failed to answer the control question and were filtered out to give the 1082 responses used in our data analysis. The order in which all questions were presented was randomized (Fig. 4 shows one example of a question).

## Results

### Visualized Emotion Ontology

The VEO was encoded in the Protégé ontology authoring tool [46] in OWL2 format. The ontology contains a total of 126 classes, 11 object and data properties, and 25 instances. We scored the quality of the VEO using OntoKeeper, a web application currently in development [47]. We compared the VEO to a sample of five cognitive ontologies (Mental State Assessment, Emotion Ontology, Mental Functioning Ontology, the Behavior Change Technique Taxonomy, and the Cognitive Atlas Ontology), which would provide us with a baseline measurement. Results of our comparison are presented in Table 4.

**Table 4:** Quality scores comparing the VEO with cognitive ontologies

Quality metrics	VEO	Cognitive ontologies (z-score)
Syntactic	0.76	0.58 (1.12)
- *Lawfulness*	1.00	0.90 (0.45)
- *Richness*	0.54	0.28 (1.68)
Semantic	0.97	0.95 (0.61)
- *Consistency*	1.00	0.96 (0.43)
- *Clarity*	0.99	0.97 (0.51)
- *Interpretability*	0.97	0.97 (0.00)
Pragmatic	0.82	0.67 (0.39)
- *Comprehensiveness*	0.82	0.67 (0.39)
Overall quality	0.85	0.68 (0.98)

For the VEO, the *syntactic* score, a score that measures the machine-readability of the ontology, based on breaches of syntax (*lawfulness* metric) and utilization of ontology features (*richness* metric), was rated at 0.76, with *lawfulness* and *richness* at 1.00 and 0.54, respectively. The *semantic* score, a score that measures the label quality of the ontology based on the consistency of labeling of concepts and instances (*consistency* metric), the ambiguity of term labels (*clarity* metric), and the meaning of ontology term labels (*interpretability* metric), was rated at 0.97, with *consistency*, *clarity*, and *interpretability* at 1.00, 0.99, and 0.97, respectively.

The *pragmatic* score, a score that assesses the utility of the ontology based on the *comprehensiveness* metric (i.e., domain coverage), was 0.82. The *overall quality score* based on equal weighting of *syntactic* (0.76), *semantic* (0.97), and *pragmatic* (0.82) scores was 0.85.

We calculated the z-scores using the data to evaluate our metrics compared to that of the sample of cognitive ontologies. The z-scores for the *syntactic*, *semantic*, and *pragmatic* metrics yielded 1.12, 0.61, and 0.39, respectively, indicating above-average machine-readability, linguistic quality, and domain coverage. Also, the z-score for the final *overall quality* was 0.98, indicating higher overall quality for the VEO than other cognitive ontologies.

Additionally, we reviewed and conferred with each other on the ontology’s veracity, and we agreed that the ontology reflected the information described in the revised OCC model. Two of the co-authors (RL, CL) have cognitive science backgrounds.

### Crowdsourced survey

In total, 1082 participants were surveyed through Amazon Mechanical Turk, and for each emotion-image pair, we determined the percentage of people that disagreed (1 or 2), were neutral (3), and agreed (4 or 5) that the image represented the emotion (Table 5).

**Table 5:** Survey results of visualization

	

Green highlights indicate statistically significant results

For the majority of the emotions (17 in total – *p* < 0.001 for 16 emotions, and *p* = 0.014 for emotion of shame), people tended to agree that our visualization matched the emotion more than they disagreed, which validates our model; these emotions included admiration, anger, fear, fears-confirmed, gratification, gratitude, happy-for, hate, hope, interest, joy, love, pride relief, satisfaction, shame, and surprise. This conclusion is based on a rigorous hypothesis testing procedure. Specifically, we assumed that the choice of each participant was distributed as a multinomial distribution with parameters *p*_1_, *p*_2_, *p*_3_ corresponding to the proportions of “Disagreed”, “Neutral”, and “Agreed”. Respectively, we then performed one-sided hypothesis tests to test whether the proportion of people who agreed is greater than the proportion of people who disagreed for each of the 25 emotions, i.e. *H*_0_:*p*_1_<*p*_3_ for each emotion. Bonferroni correction was applied to control the family-wise error rate at 5%.

*P*-values were reported in Table 5. In statistical hypothesis testing, *p*-value is a probability value which quantifies the evidence from the data to support the alternative hypothesis against the null hypothesis. A smaller *p*-value indicates strong evidence against the alternative hypothesis. A critical value is a cut-off of the *p*-value to determine whether to reject the null hypothesis. Here in this study, the alternative hypothesis is that the proportion of participants agreed is greater than the proportion of participants disagreed while the null hypothesis is that they are equal. Accounting for the multiple testing, we reject the null hypothesis for *p*-value less than 0.002. Significant results of higher proportion of agreed than disagreed (*p*<0.001) were found for 16 out of 25 emotions including all of the emotions previously stated except for shame (*p*=0.014).

For the remaining eight emotions, more people disagreed than agreed with our visualization. However, for five of these emotions, including disappointment, disgust, gloating, pity, and remorse, more people agreed with our emotion-image pairs than they did for the incorrect emotion-image pairs. In these cases, the randomly-selected incorrect emotion-image pairs included disappointment-interest, disgust-satisfaction, gloating-gratitude, pity-admiration, and remorse-gratification, respectively. For distress, reproach, and resentment, however, more people agreed with the incorrect emotion-image pairs than they did with the correct ones; these incorrect pairs included distress-fear, reproach-resentment, and resentment-disappointment, respectively.

## Discussion

In the future, we could expand the VEO by creating nuances within certain emotion types–for instance, fear-like states can range from those that are mild (e.g., concern) to those that are intense (e.g., terror). These types of states could be included as subclasses in the ontology. We also intend to expand the terminological space with some of the affective terms found in WN-AFFECT. Additionally, we could add instances in the future that represent an individual user’s emotions.

Overall, the survey results validated the accuracy of our emotion visualizations. More people agreed than disagreed that the image matched the emotion displayed for 17 emotions (with 16 out of these 17 emotions found to be statistically significant), and vice versa for eight emotions. However, only for the three emotions of distress, reproach, and resentment did people prefer the incorrect emotion-image pair to the correct one. One reason the incorrect emotion-image pair was preferred for distress could be due to its name—-distress and sadness have slightly different connotations, and if we had used the name “sadness”, perhaps the percentage of people that agreed with our visualization would be higher. After all, even though people thought that the image for fear represented distress (in the incorrect emotion-image pair), they still confirmed that the image for fear was accurate at a high rate (65.0%).

Additionally, in future studies and from the findings of the survey results, it would be helpful to further investigate the eight emotions that did not support our visualizations by comparing them to different incorrect emotion-image pairs. This could allow us to understand whether the specific randomly-chosen incorrect pair had any influence on our results or if they still hold with different pairs used. If so, these results can inform us in regard to editing our visualizations so that they are more representative of each emotion. Our research also does not consider the use of motion, which could enhance the visualizations in the future.

**Fig. 5:**
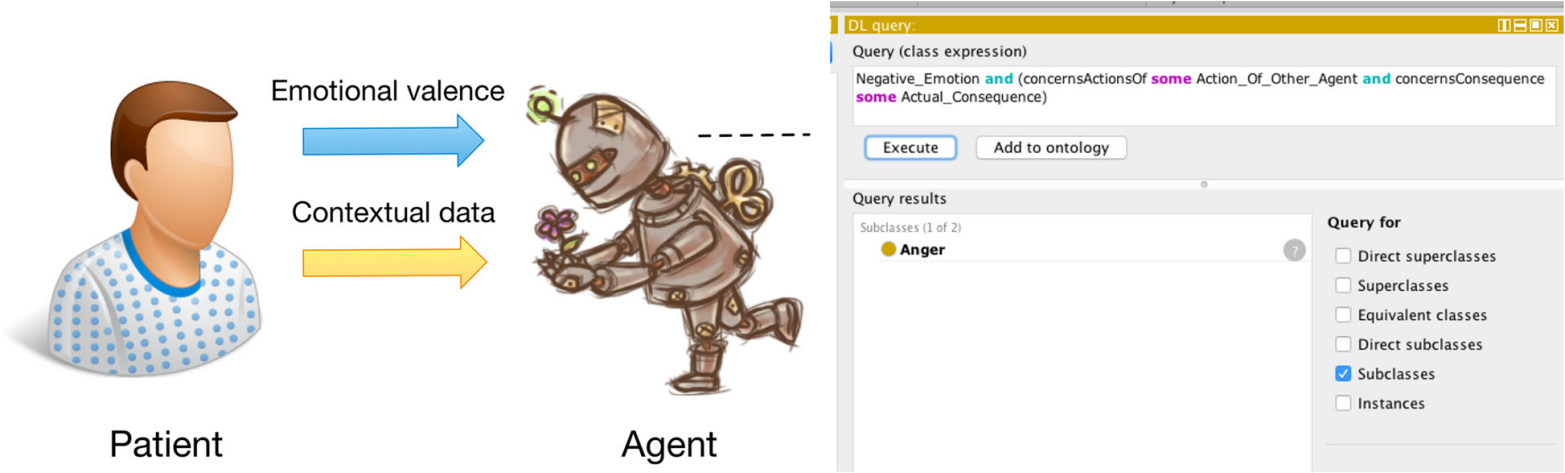
Utilization of the VEO and the processing of expression information to infer emotion of the patient. “People Patient Male Icon” by Icons-Land [48], and “Steampunk Robot Icon” by mirella.design [49] - licensed free for non-commercial use

This study will permit machines to utilize the VEO to interpret and understand emotions, with the purpose of improving interaction with human users, such as patients. For clarification, recall that ontologies are artifacts of encoded knowledge to help machines understand domain concepts and the relationships between them. Codifying affective knowledge would help intelligent agents, specifically conversational agents, to understand the underlying emotions during their interactions with humans. Looking at an emotion like love, which according to the OCC model, contains positive emotional valence involving the appraisal of some aspect of an object, or anger, which contains negative emotional valence relating to someone’s actions and the subsequent outcomes of the actions. A software agent can potentially capture contextual information and emotional valence data, and through the use of descriptive logic queries, reason what the user is feeling or expressing (see Fig. 5). The use of ontologies to define emotions for machines and then comprehend the emotions of users makes this possible. Further research could investigate processing of the user’s emotions from utterances or other modalities of expression. This would also include developing the software that interfaces with the ontology and employing it in conversational agents.

## Conclusion

Based on metrics for ontology evaluation, the Visualized Emotion Ontology (VEO) revealed to have better domain coverage, machine readability, and linguistic quality than the selected cognitive ontologies from the BioPortal. The VEO also links to composite visualizations, based on published research, that expressed each emotion defined in the VEO. From the Amazon Mechanical Turk survey we conducted, we determined that the majority of the visualizations accurately represented their emotions, validating our model.

The genesis of this work was to provide a means to enhance the patient-provider communication for patient education by defining emotions for machines. Specifically, conversational agents assisting physicians for vaccine counseling could augment the experience by emoting through visualizations to enhance the synthesized, deadpan utterances. This would serve as alternative to more complex and resource expensive options like avatars or computer-generated faces. The visualized emotions and the VEO could presumably be utilized in other applications that involve human computer interaction.

## Abbreviations

ACC: Anterior cingulate cortex
BFO: Basic formal ontology
EMO: The emotion ontology
HEO: Human emotion ontology
HIT: Human intelligence task
MTurk: Amazon mechanical turk
OCC: Ortony, clore, & collins
OMD: Ontology of mental disease
OWL: Web ontology language
OWL2: Web ontology language, version 2
RDF: Resource description framework
STG: Superior temporal gyrus
VEO: Visualized emotion ontology
W3C: World wide web consortium

## Footnotes

1 The Ekman emotions and the revised OCC model list of emotions overlapped, except for one emotion, surprise, which was added to VEO.

2 With approval by the University of Texas Health Science Center’s Committee for the Protection of Human Subjects – HSC-SBMI-17-0641
